# Molecular signatures of epithelial oviduct cells of a laying hen (*Gallus gallus domesticus*) and quail (*Coturnix japonica*)

**DOI:** 10.1186/s12861-018-0168-2

**Published:** 2018-04-04

**Authors:** Katarzyna Stadnicka, Anna Sławińska, Aleksandra Dunisławska, Bertrand Pain, Marek Bednarczyk

**Affiliations:** 1 Department of Animal Biochemistry and Biotechnology, UTP University of Science and Technology, Mazowiecka 28, 85-084 Bydgoszcz, Poland; 2 University of Lyon, Université Lyon 1, INSERM, INRA, Stem Cell and Brain Research Institute, U1208, USC1361, Bron, France

**Keywords:** Laying hen, Laying quail, Oviduct, Epithelial cells, Progenitor cells, Molecular signatures

## Abstract

**Background:**

In this work we have determined molecular signatures of oviduct epithelial and progenitor cells. We have proposed a panel of selected marker genes, which correspond with the phenotype of oviduct cells of a laying hen (*Gallus gallus domesticus*) and quail (*Coturnix japonica*). We demonstrated differences in characteristics of those cells, *in tissue* and in vitro*,* with respect to different anatomical and functional parts of the oviduct (infundibulum (INF), distal magnum (DM, and proximal magnum (PM)). The following gene expression signatures were studied: (1) oviduct markers (estrogen receptor 1, ovalbumin, and SPINK7 - ovomucoid), (2) epithelial markers (keratin 5, keratin 14, and occludin) and (3) stem-like/progenitor markers (CD44 glycoprotein, LGR5, Musashi-1, and sex determining region Y-box 9, Nanog homebox, OCT4/cPOUV gene encoding transcription factor POU5F3).

**Results:**

In chicken, the expression of oviduct markers increased toward the proximal oviduct. Epithelial markers keratin14 and occludin were high in distal oviduct and decreased toward the proximal magnum. In quail oviduct tissue, the gene expression pattern of oviduct/epithelial markers was similar to chicken. The markers of progenitors/stemness in hen oviduct (Musashi-1 and CD44 glycoprotein) had the highest relative expression in the infundibulum and decreased toward the proximal magnum. In quail, we found significant expression of four progenitor markers (LGR5 gene, SRY sex determining region Y-box 9, OCT4/cPOUV gene, and CD44 glycoprotein) that were largely present in the distal oviduct. After in vitro culture of oviduct cells, the gene expression pattern has changed. High secretive potential of magnum-derived cells diminished by using decreased abundance of mRNA. On the other hand, chicken oviduct cells originating from the infundibulum gained ability to express *OVM* and *OVAL*. Epithelial character of the cells was maintained in vitro*.* Among progenitor markers, both hen and quail cells expressed high level of SOX9, LGR5 and Musashi-1.

**Conclusion:**

Analysis of tissue material revealed gradual increase/decrease pattern in majority of the oviduct markers in both species. This pattern changed after the oviductal cells have been cultured in vitro. The results can provide molecular tools to validate the phenotype of in vitro biological models from reproductive tissue.

**Electronic supplementary material:**

The online version of this article (10.1186/s12861-018-0168-2) contains supplementary material, which is available to authorized users.

## Background

### Avian oviduct in biomedical research

Avian species are excellent biological models in reproduction and tumorigenesis [1] as well as efficient source of secreting cells for use in bioreactors [2–4]. Both hen and quail oviduct cells secrete human therapeutic proteins after genetic modification [3]. Therefore the oviduct epithelium is a useful and fast in vitro model to test for the efficiency of viral [5] and nonviral genetic constructs [6] to study the modified secretome. Both quail and hen produce cellular substrates for the development of vaccines [7]. Genetic markers, including markers of stemness, are useful to identify mechanisms of malignant changes in a fallopian tube, because the somatic stem cells contribute to a population of tumor-initiating cells [8]. Recently, the knowledge about cell differentiation, physiology, and cancerogenic changes in avian oviduct has been extrapolated to women fallopian tube and uterine tract [9, 10]. However, in avian species, markers of stemness in oviduct cells have not been reported yet. There is a knowledge gap regarding distinctive features of the epithelial cells in in vitro conditions vs. their status in tissue, which limits full understanding and characterization of this cellular model. In this paper, we have made initial attempts to confirm progenitor molecular signatures in oviducts of laying hen (*Gallus gallus domesticus*) and quail (*Coturnix japonica*), both in tissue and in cultured oviduct epithelial cells (in vitro assay). We have addressed the following questions: What is the location of progenitor cells in avian oviduct tissue? What is an individual molecular characteristic of distal oviduct tissue compartments? Is this distinctive characteristic stable once the cells are plated in in vitro condition? Is the molecular pattern shared between these two model species (laying hen and quail) used for oviduct studies? Altogether, this study aims to provide a new understanding of molecular characteristic of oviduct epithelial cells in avian species.

### Adult epithelial cells in the oviduct

In adult tissue, epithelial progenitor cells have limited potential to divide and they can develop only into few differentiated cell types. They express stem cell markers and can differentiate into epithelial cells with various phenotypes.

Mucous epithelium of an avian oviduct is composed of simple columnar cells equipped with cilia to move the ovum from distal to proximal oviduct and of nonciliated secreting cells. Both cell types require sustained renewal from the stem cell compartment and a high proliferation and maturation activity from the progenitor compartment. Those compartments are putatively based under the luminal epithelium as cellular niches [11]. In a mammalian fallopian tube, stem cells niches were tracked using antibodies and genetic markers and were found to be localized in the distal fallopian tube [8, 12]. In our earlier research, we determined faster proliferation of cultivated hen oviduct cells derived from infundibulum/distal magnum compared to the cells that were sourced from a proximal magnum. We have also determined that distal oviduct compartments were positively immunostained against CD44 and p63, which are known to be epithelial stem/progenitor markers [13]. Thereby, we have hypothesized that distal segments of avian oviduct contain progenitor gene expression signatures.

### Genetic markers of distinctive signatures in avian oviduct epithelium

**Fig. 1:**
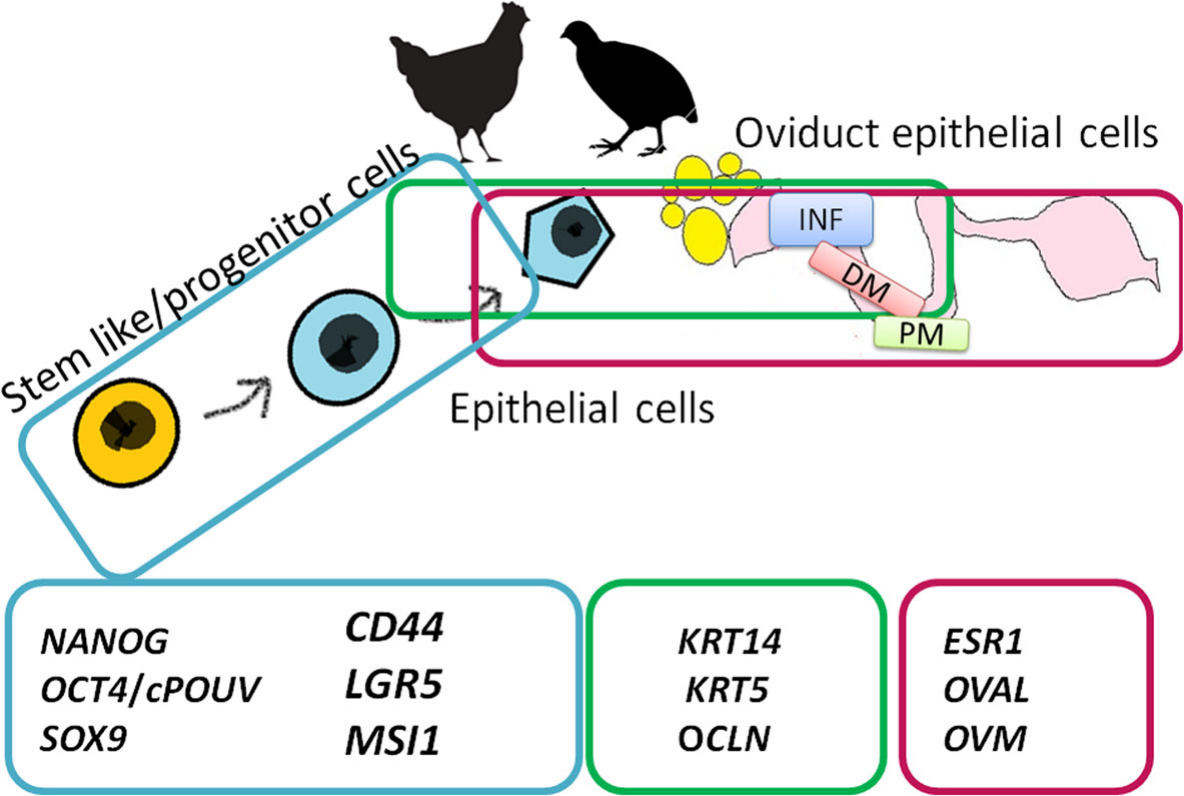
A graphical representation of selected panel of epithelial genetic markers associated with oviduct cells. Three panels of epithelial genetic markers were proposed to provide a pattern of molecular signatures in the oviduct of hen and quail in 3 compartments: INF – infundibulum, DM – distal magnum, PM – proximal magnum. The first panel shown in the picture refers to stem-like markers: Nanog homebox (NANOG), octamer-binding protein 4 (OCT4/cPOUV) and sex determining region Y-box 9 (SOX9); and epithelial progenitor cells: cell surface glycoprotein CD44, leucine-rich repeat containing G protein-coupled receptor 5 (LGR5), and Musashi-1 (MSI-1). The second panel refers to epithelial cells: keratins KRT 5 and 14 and occludin (OCLN). The third panel refers to functional avian oviduct cells: estrogen receptor-1 (ESR1), ovalbumin (OVAL) and ovomucoid (OVM)

Characterization of oviduct cells using molecular markers for epithelial progenitors contributes to the understanding of differentiation and regeneration processes, which occur in the oviduct epithelium. As reported earlier, the self-renewal activity of cells in the fallopian tube occurs in its distal part [14, 15]. Thereby, in this paper, we have focused on distal parts of the oviduct (the closest to ovaries and abdomen) to follow the molecular characteristics of the cells *in tissue* and in vitro. We propose a panel of epithelial genetic markers to determine the progenitor/epithelial cell pattern in selected compartments of the oviduct (Fig. 1). In particular, we have aimed to reveal which of the avian oviduct compartments (infundibulum (INF), distal magnum (DM), or proximal magnum (PM)) carry known progenitor signatures*.*

## Methods

### Isolation of the oviduct tissue

In this study, all the procedures involving experimental animals were approved by the Local Ethics Committee for Animal Research (http://lke.utp.edu.pl) located at the Faculty of Animal Breeding and Biology, UTP University of Science and Technology in Bydgoszcz (study approval reference number 35/2012, in accordance with the 2010_63_UE_PL Directive). The Hybrid Tetra SL laying hens (*n* = 6, 40 weeks old) were obtained from a commercial farm (Nowosc, Pradocin, Poland). Laying Japanese quails (*n* = 6, 10 weeks old) were obtained from a commercial producer (K. Drazek, Wyzne, Poland). All birds laid eggs at a daily rate and no hormonal stimulation was applied for this study. Immediately upon transportation, the animals were sacrificed by cervical dislocation. Only the oviducts after egg passage, with ovum position in a shell gland, were used for the experiments. Each oviduct was rinsed twice in tube filled out with 25 mL physiological buffered saline (PBS) w/o Mg, w/o Ca (Lonza Biosciences, Celllab, Warszawa, Poland), which was gently mixed with Penicillin-Streptomycin solution at 1:100 (v:v; Life Technologies, Warszawa, Poland).

### In vitro culture of oviduct epithelial cells

The epithelial cells were isolated from the oviduct tissue using the methodology described earlier [16]. Immediately after tissue collection, three oviduct fragments were dissected: infundibulum, distal magnum, and proximal magnum, each 3 cm long. Each fragment was cleaned off the mesentery tissue and minced with a scalpel blade on a Petri dish. The minced fragments were digested in a solution of 1 mg/mL collagenase P (Sigma-Aldrich, Poznan, Poland) in Advanced Dulbecco’s Modified Eagles Medium-F12 (DMEM/F-12; Life Technologies, Warszawa, Poland) for 30 min at 37 °C, on a shaker. Due to the size of oviductal tubes, the amount of minced tissue was ~ 50% less in quail than in hen. Thus, adequately lower volumes of digestion solution were applied to process the quail oviduct tissue. The cells were counted manually using Neubauer hemocytometer and seeded at a density of 4 × 10^4^ cells/cm^2^ into 25 cm^2^ vented BD type Primaria flasks (Becton Dickinson, Diag-med, Warszawa, Poland). The cells were incubated in 7% CO_2_ atmosphere at 37 °C. The oviduct cells were maintained in DMEM-F12 supplemented with 5% (*v*/v) fetal bovine serum (FBS; Life Technologies, cat. 16,140-063, batch No. 41G4541K, Warszawa, Poland), 1% (v/v) nonessential amino acids (Sigma-Aldrich, Poznan, Poland), 20 mM L-glutamine (Sigma-Aldrich, Poznan, Poland), 10 ng/mL human epidermal growth factor (human EGF; R&D Bioscience, Biokom, Janki, Poland), 1% (v/v) antibiotic – antimycotic solution (Life Technologies, Warszawa, Poland), 0.5 μg/mL hydrocortisone (Sigma Aldrich, Poznan, Poland) and 5 μg/mL insulin-transferrin-selenium (ITS; Sigma-Aldrich, Poznan, Poland). The viability and the proliferation of oviduct cells were measured by a real-time cell analyzer (RTCA) supplied by xCELLigence system (Roche Applied Science, Basel, Switzerland). The measurements of proliferating cells were conducted at 3.1 h intervals through 287 h post seeding, in accordance with the producer’s manual. The cells intended for sampling were cultivated for 5–7 days prior to harvesting and analysis. Every second day, the epithelial colonies were counted and photographed under an objective with phase contrast (Zeiss Axiovert 40) equipped with a digital camera (Canon EOS 600). The cells were harvested upon reaching 80% of growth confluence. Cultured oviduct epithelial cells were referred as chicken oviduct epithelial cells (COEC) or quail oviduct epithelial cells (QOEC) in further parts of this paper.

### RNA isolation from oviduct tissue, COEC, and QOEC

RNA was isolated from three different sections of the oviduct tube (INF, DM, and PM) and cultivated oviduct cells, derived from the respective birds. For in vivo assay, INF, DM, and PM fragments, each 1 cm long, were cut off aseptically and put separately into Eppendorf tubes containing 3.0 mL RNAfix (EURx, Gdansk, Poland). Tissue samples were kept for 24 h at 4 °C and subsequently stored at − 20 °C until isolation of RNA. For RNA isolation from COEC, confluent cells were detached using Accutase® solution (A&E Life Sciences, Gentaur, Sopot, Poland) and centrifuged at 220×*g* for 5 min at room temperature (RT). Cell pellets were resuspended in 0.5 mL RNAfix (EURx, Gdansk, Poland) to preserve cells prior to RNA isolation. RNA was extracted using the universal RNA purification kit (EURx, Gdansk, Poland) according to manufacturer’s recommendation. RNA was quantified using spectrophotometry and RNA quality by gel electrophoresis.

### RT-qPCR analysis

Reverse transcription was performed with Maxima First Strand cDNA synthesis kit for RT-qPCR (Thermo Scientific/Fermentas, Vilnius, Lithuania). cDNA was diluted to a final concentration of 70 ng/μL and stored at −20°C. Reverse transcription-quantitative polymerase chain reaction (RT-qPCR) was performed in a total volume of 10 μL, which included Maxima SYBR Green qPCR Master Mix (Thermo Scientific/Fermentas, Vilnius, Lithuania), 1 μM of each primer (forward and reverse), and 2 μL of diluted cDNA (140 ng). Primer sequences (Table 1) were derived from the literature or designed with NCBI Primer Blast, based on cDNA reference sequences [17]. Thermal cycling was conducted in LightCycler II 480 (Roche Applied Science, Basel, Switzerland). qPCR thermal profile consisted of initial denaturation at 95 °C for 20 min, followed by 40 cycles of amplification including 15 s of denaturation at 95 °C, 20 s of annealing at 58 °C, and 20 s of elongation at 72 °C. After completion of the amplification reaction, a melting curve was generated to test for the specificity of RT-qPCR. For this purpose, the temperature was gradually increased to 98 °C with continuous fluorescence measurement.

**Table 1:** Primer sequences used in RT-qPCR study

Gene	Forward (F) and reverse (R) primers (5′ ➔ 3′)	Amplicon size (bp)	Genome	Reference^a^
*CD44*	F: ACGAGGAGCAAAGCATGTGA R: GTGAGCCGTCCTCATTGTCA	94	A	[6]
*CD44*	F: CGGAGTACTGAGGGCATCAC R: TGACTGTTGTGATGATGGTGGT	133	B	this study
*ESR1*	F: CAGGCCTGCCGACTAAGAAA R: GGTCTTTCCGGATTCCACCT	64	A	this study
*ESR1*	F: CAGGCCTGCCGACTAAGAAA R: CTGGACTCCTGCTCCTCTCT	119	B	this study
*KRT5*	F: GGGTGTTGGAGCCGTGAGTGTC R: TGCCAAGACCACTGCCCATGC	137	A	[26]
*KRT14*	F: GCGAGGACGCCCACATCTCTTC R: TGAGCGCCATCTGCTCACGG	150	A	[26]
*LGR5*	F: GAAATGCTTTGATGGGCTCC R: TGATAGCAGTGGGGAACTCG	80	A	this study
*LGR5*	F: AACCAACTACGCCAGGTTCC R: CATCCAGGCGTAGAGACTGC	70	B	this study
*MSI1*	F: TTCGGGTTCGTCACGTTCAT R: TCGTTCGGGTCACCATCTTG	139	A	this study
*MSI1*	F: AGTACTTCAGCCAGTTCGGC R: CCTTCGGGTCAATCTGGATCT	83	B	this study
*NANOG*	F: TGCACACCAGGCTTACAGCAGTG R: TGCTGGGTGTTGCAGCTTGTTC	120	A	[26]
*NANOG*	F: TCTACCACAGAGCGGGTTTC R: CCCATTCCCGTAAGTCTGGC	148	B	this study
*OCLN*	F: GAGGAGTGGGTGAAGAACGTG R: GGTGCCCGAGGGGTAGTA	150	A	this study
*OCLN*	F: TCCCGGCTGCCATTTTAAGG R: GAACATGGTGAACCTCCGCC	50	B	this study
*OCT4/* *cPOUV*	F: TGCAATGCAGAGCAAGTGCTGG R: ACTGGGCTTCACACATTTGCGG	114	A	[26]
*OVAL*	F: CGTTCAGCCTTGCCAGTAGA R: AGTATTCTGGCAGGATTGGGT	60	A	this study
*OVM*	F: TATGCCAACACGACAAGCGA R: CCCCCTGCTCTACTTTGTGG	133	A	this study
*SOX9*	F: GAGGAAGTCGGTGAAGAACG R: GCTGATGCTGGAGGATGACT	124	A	[36]
*SOX9*	F: CAGCAAGAACAAACCCCACG R: TTCAACAGCCTCCACAGCTT	147	B	this study
*ACTB*	F: CACAGATCATGTTTGAGACCTT R: CATCACAATACCAGTGGTACG	101	A	[37]
*UB*	F: GGGATGCAGATCTTCGTGAAA R: CTTGCCAGCAAAGATCAACCTT	147	A	[38]

^a^Primer sequences reported in this study were designed based on the cDNA reference sequence and NCBI Primer Blast [17]. Oligonucleotide primers spanned exon–exon boundaries to avoid unspecific gDNA amplification. Genome A – chicken (*G. gallus*)*,* B – quail (*C. japonica*)

### Relative quantification of gene expression

Relative gene expression analysis was performed for each experimental group with ∆∆Ct method [18], using Ubiqutin C (*UB*) and β-actin (*ACTB*) as reference housekeeping genes. Geometric means of Ct value of both reference genes was used in calculations. For tested samples, ΔCt was calculated by subtracting mean Ct values of the reference genes from Ct values of the target gene. A base sample (calibrator) was defined by an origin different from the reproductive system. For *in tissue* study, muscle samples from the same birds were used. For in vitro study, the chicken macrophage-like cell line [19] was used as a calibrator. ΔΔCt was then calculated using the equation: ΔCt sample – ΔCt calibrator. Fold change of the gene expression was calculated as: *R* = 2^–ΔΔCt^.

### Statistical analysis

RT-qPCR results were statistically analyzed using SAS Enterprise Guide 6.1 (SAS Institute, Cary, NC, USA). All tests were conducted on ΔCt values. First, Shapiro-Wilk test was used to assess the normality of data distribution. Then the significance of changes in the gene expression (in comparison to calibrator samples) was conducted by Student’s *t*-test (*P* < 0.05). Finally, multiple comparisons for all pairs (e.g., oviduct fragments or donor species) were performed with one-way ANOVA followed by Tukey’s HSD post hoc test. Standard error of the mean (SEM) was used as a parameter of variability within the group.

## Results

### Primary cultures of hen and quail oviduct epithelial cells

**Fig. 2:**
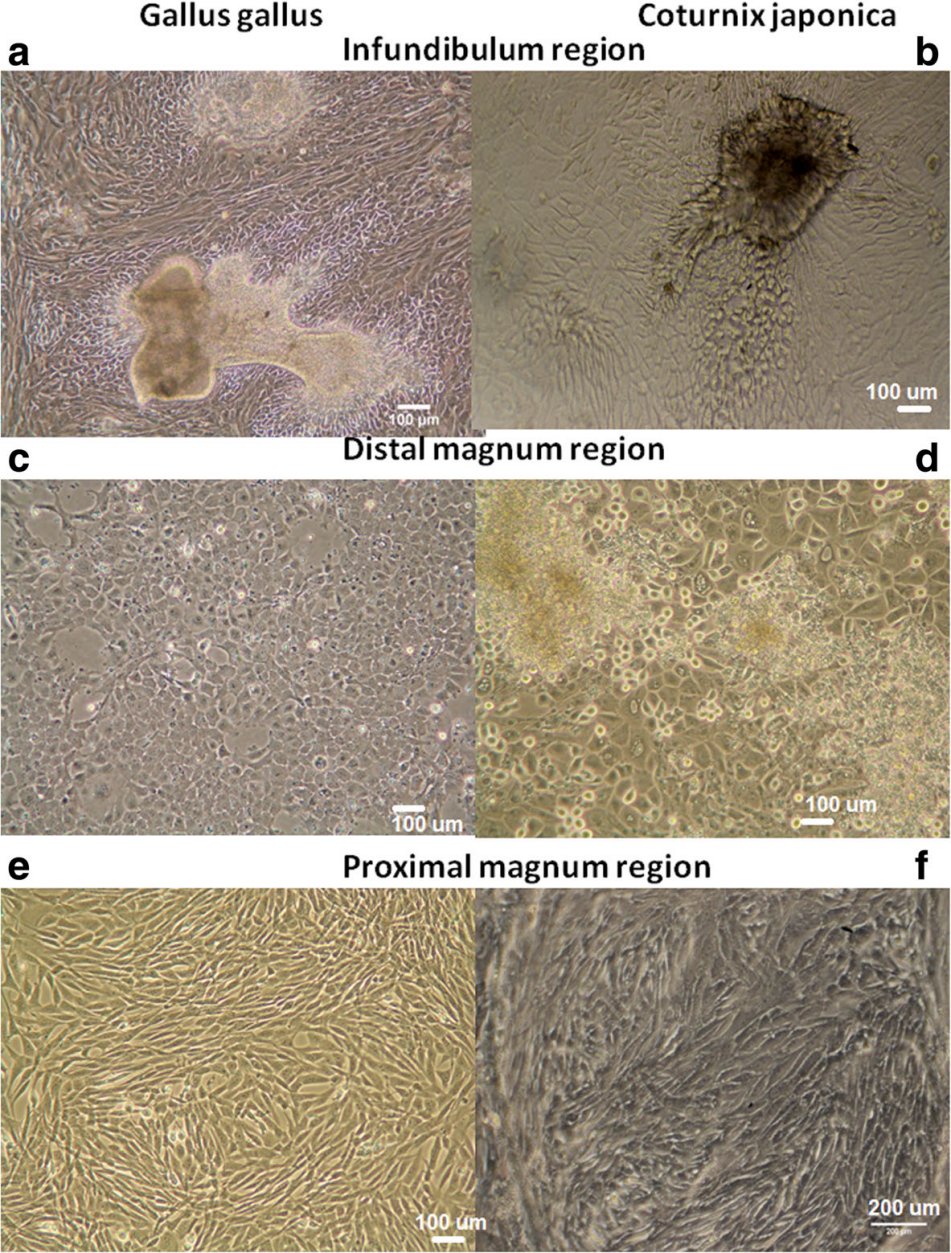
Phenotypes displayed by hen and quail oviduct cell colonies in vitro*.* **a**–**b**: confluent monolayers and visible spheres of colony-initiating cells isolated from the region of infundibulum neck (INF); magnification: × 100. **c**–**d**: confluent monolayers of epithelial cells isolated from distal magnum (DM), showing typical cobble-like morphology; magnification × 100. **e**–**f**: confluent epithelial monolayer, typically observed in cultivated cells that are originating from the oviduct magnum, showing mostly fibroblast-like morphology; magnification × 100. In each case, the cells were seeded at a density of 4 × 10^4^ cells/cm^2^


Cultivated oviduct cells of hen (COEC) and quail (QOEC) reached the confluence after 5–7 days after seeding. The COEC and QOEC isolated from the infundibulum region typically occurred as cellular spheres, which attached to the polystyrene culture vessel after 3 days post seeding and were consequently creating epithelial-like colonies, which spread on the surface of the culture vessel. Once the small epithelial colonies appeared beneath the spheres, they enter a high proliferation phase to rapidly form a confluent monolayer. Typical cultures from the infundibulum region were characterized by numerous compact epithelial islands, oval in shape, surrounded by elongated cells of mesenchymal or fibroblast-like phenotype (Fig. 2a, b). Epithelial cells isolated from the region of distal magnum (a transition region between infundibulum and proximal magnum) formed visible epithelial islands 3 days after seeding, which was sooner compared to the infundibulum region. In the case of distal magnum, spheres that formed epithelial-like colonies in vitro were half the size of those isolated from the infundibulum and about two times less colonies were initiated, compared to those from the infundibulum region on day 3 (Fig. 2c, d). In most cases, the epithelial colonies from the distal magnum proliferated fast and were ready for passage by 6–7 days after seeding. The cells from the region of proximal magnum usually did not form spheres in the beginning of the cultivation (Fig. 2e, f). Typically, in proximal magnum, the proliferating epithelial colonies were observed in 3–5 days post seeding. The microscopic observations of growing colonies were in line with the measurements acquired from the xCELLigence real-time cell monitoring system. The peak of the proliferation was determined for the cells from all oviduct compartments after 3 days post seeding: at 78.27 h for the INF part, at 79.05 h for the DM part, and at 79.05 h for the PM part. Then, the cell proliferation entered the plateau, which lasted 16.22 h for INF cells, 15.5 h for PM cells, and 10.85 h for DM cells. The cells from PM displayed larger morphology than cells from INF, and the shape of colonies was not compact, but oval and irregular. The confluent monolayer was heteromorphic, consisting of epithelial and fibroblast-like colonies (Fig. 2e, f). Motile cilia, which are characteristic for oviduct ciliated cells, were observed in the cultivated primary colonies, but only until the first passage. A movie file shows this in more detail (Additional file 1).

Additional file 1: Visualization of a typical phenotype of cultivated oviductal epithelial cells. The recording of the cultivated oviduct epithelial cells allows one to follow the typical cobble-like structure of lining epithelial cells and the rotatory movement of cilia on the nonsecreting ciliated cells, which are coisolated with the secreting tubular gland cells. (MP4 12,229 kb)


### Gene expression analysis

In this study, we have used three gene panels to characterize oviduct fragments of hen and quail, and the respective primary epithelial cell cultures that were derived from them. Those panels were comprised of oviduct (*ESR1*, *OVAL*, and *OVM*), epithelial (*KRT5, KRT14*, and *OCLN*), and stem-like/progenitor (*LGR5*, *MSI1*, *SOX9*, *NANOG*, and *OCT4*/*cPOUV*) gene expression signatures. Table 2 presents the overview of the gene function and the sequence similarity between a hen and a quail.

**Table 2:** List of genes with functional annotations proposed as markers of avian oviduct epithelial cells

Gene symbol/	Gene name	Species/ NCBI reference	Gene function (Gene atlas)	Biological process	Reference (Function)	% of identity with human protein sequence	% of identity with quail genomic sequence^a^
ESR1	estrogen receptor 1	*G. gallus* 396,099	Encodes the protein estrogen receptor alpha, plays role in the sex differentiation of reproductive tract, regulates the expression of oviduct genes	4A- nuclear receptor; transcription regulator; binding estradiol, epithelial cell development, cell differentiation	[34, 39]	79	99
		*C. japonica* 107,311,566		Transcription regulator; binding estradiol, epithelial cell development, cell differentiation	[40]	n/a	
OVAL	Ovalbumin-SERPINB14	*G. gallus* 396,058	Encodes ovalbumin in Ov-serpin family, located in extracellular space	Oviduct secretome, binds calcium, responds to steroid hormones, constitutes egg white	[41]	41	93
		*C. japonica* 107,309,565		Oviduct secretome, constitutes egg white, responds to steroid hormones	[42]	n/a	
OVM	SPINK7- serine peptidase inhibitor, Kazal type 7	*G. gallus* 416,236	Encodes ovomucoid protein to the extracellular space, responds to steroid hormones, binds IgE, IgG	Secreted as egg white protein, allergenic as a food component, responsive to steroid hormones (progesterone), allergenic component of an egg white	[43, 44]	44	96
		*C. japonica* 107,320,484				n/a	
KRT5	keratin 5	407,779	Encodes protein keratin 5, type II cytoskeletal 5	Interacts with KRT14 to form cytoskeleton of basal epithelium, expressed in stem cells of fallopian tube, epithelial differentiation	[15]	80^b^	98
KRT14	keratin, type I cytoskeletal 14	408,039	Marker of the stratified epithelium as keratin filament	Interacts with KRT5 to form the cytoskeleton of basal epithelium, expressed in tumor cells of fallopian tube, marker of chicken keratinocytes, epithelial differentiation	[26]	69	100
OCLN	occludin	*G. gallus* 396,026	Encodes protein occludin, marker of tight junctions in epithelial cells	Component of plasma membrane, role in cellular binding, forms tight junctions	[26]	47	96
		*C. japonica* 107,325,447				n/a	
CD44	cell surface glycoprotein CD44	*G. gallus* 395,666	Encodes CD44 antigen, marker of epithelial stem cells in fallopian tube	Role in cell adhesion (cell to cell) and postponement of the apoptotic process	[15]	48	100
		*C. japonica* 107,314,385				n/a	
LGR5	Leucine-rich repeat containing G protein-coupled receptor 5	*G. gallus* 427,867	Encodes protein LGR5, induced by Wnt/β-Catenin signaling	Marker of stem cells in the ovary and tubal epithelia	[14]	30^c^	99
		*C. japonica* 107,310,333				n/a	
MSI-1	Musashi-1; Musashi RNA binding protein- 1	*G. gallus* 416,979	Marker of epithelial early lineage, marker of stem cells in human endometrium	Maintains proliferation and multipotent potential of epithelial cells (emerging from Müllerian duct)	[45]	80	100
		*C. japonica* 107,321,418			[31]	n/a	
NANOG	Nanog homebox	*G. gallus* 100,272,166	Encodes transcriptional factor of pluripotency: homebox protein NANOG, Marker of stem cells in ovarian epithelium	Maintains stem cell population, chicken stem cell marker	[32]	89	100
		*C. japonica* 107,318,297			[46]	n/a	
OCT4/ cPOUV	POU domain class 5 transcription factor 3, Octamer-binding protein 4	427,781	Encodes transcription factor POU5F3	Maintains cell pluripotency, maintains population of somatic stem cells, shows responses to wounding	[33]	43	97
SOX9	SRY sex determining region Y-box 9	*G. gallus* 374,148	Encodes HMG box transcription factor, marker of epithelial early lineage, transcription epithelial-mesenchymal transition marker	Negatively regulates the differentiation of epithelial cells, maintains the population of somatic stem cells, plays role in transdifferentiation; regulation of cell adhesion, activated during chondrogenesis in chicken	[28]	99	100
		*C. japonica* 107,322,214			[29]	n/a	

^a^Blasted with http://viewer.shigen.info/uzura/blast_result.php. ^b^similarity for gene is given; no protein sequence of protein KRT5 is available for *G. gallus*. ^c^UNIPROT blasting tool shows for only 30% identity of a *G. gallus* sequence with human LGR5, but the same protein sequence shows 95% identity with human VAV3 GDP/GTP exchange factor. For a quail, only 900 proteins are annotated in existing UniProt databases. Thus, when a gap in quail database [22] limits the interpretation of a sequence, a relevant genomic alignment onto chicken was performed [23]. Depending on the database used (ENSEMBL, NCBI, and/or UniProt), sequences of the genes selected for this study had 89%–100% similarity. Thereby, gene expression assays developed were comparable between both species

The overall gene expression of the markers analyzed in both species (hen and quail) and sample types (tissue and in vitro) is presented in Table 3. All twelve genes were expressed only in COEC. Ten out of twelve genes were expressed in oviduct tissues—sourced from both hen and quail. In the hen tissue, two progenitor markers (*LGR5* and *OCT4*/*cPOUV*) were at a level too low to be detected. In the quail tissue, one epithelial marker (*OCLN*) and one progenitor marker (*LGR5*) were not detected. In QOEC, *OVAL* and *OVM* (oviduct markers) were not expressed as well as *OCLN* (epithelial marker). In both species, *LGR5*, a progenitor marker, was absent in the oviduct tissue, but then we detected it in the oviduct epithelial cell culture. *OCLN* was not expressed in quail oviduct—neither in the tissue, nor in the cell culture.

**Table 3:** Expression of the oviduct, epithelial, and progenitor markers in oviduct tissue and cultured oviduct epithelial cells in hen and quail

Gene panel	Gene	Hen		Quail	
		Tissue^a^	Cell culture^b^	Tissue^a^	Cell culture^b^
Oviduct markers	*ESR1*	+	+	+	+
	*OVAL*	+	+	+	ND
	*OVM*	+	+	+	ND
Epithelial markers	*KRT5*	+	+	+	+
	*KRT14*	+	+	+	+
	*OCLN*	+	+	+	+
Stem-like/Progenitor markers	*CD44*	+	+	+	+
	*LGR5*	ND	+	+	+
	*MSI1*	+	+	+	+
	*SOX9*	+	+	+	+
	*NANOG*	+	+	+	+
	*OCT4/cPOUV*	ND	+	+	+

^a^Hen/quail oviduct tissue, divided into three fragments: *INF* infundibulum, *DM* distal magnum, and *PM* proximal magnum; ^b^Hen/quail oviduct epithelial cell culture derived from different parts of the oviduct (INF, DM, or PM) and cultured in vitro; “+” denotes positive result of RT-qPCR analysis (Ct < 35), meaning that the gene was expressed in a given sample. *ND* not detected

### Characterization of gene expression signatures in hen and quail oviduct tissue

**Fig. 3:**
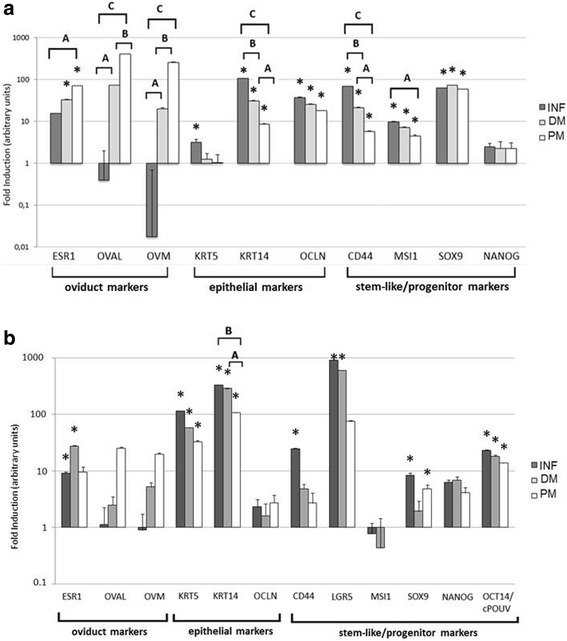
Expression of oviduct, epithelial, and progenitor markers in different fragments of hen (**a**) and quail (**b**) oviduct tissue. Relative gene expression analysis was conducted with RT-qPCR method in three oviduct fragments: infundibulum (INF), distal magnum (DM) and proximal magnum (PM). Pairwise t-test was conducted to determine the significant modulation of the gene expression in the oviduct as compared to the external calibrator (breast muscle) (*P* < 0.05). An asterisk (*) indicates that the gene is differentially expressed, compared to the calibrator. Letters A, B, and C in brackets indicate results of one-way ANOVA multiple comparisons between different fragments of the oviduct (*P* < 0.05)

Hereby we have characterized gene expression profile in different parts (INF, DM, and PM) of hen and quail oviduct tissue (Fig. 3). In hen oviduct (Fig. 3a), the expression of oviduct markers (*ESR1*, *OVAL* and *OVM*) increased spatially, from distal to proximal part of the oviduct with a peak in PM (*P* < 0.05). Reversely, the expression of epithelial markers, *KRT14* and *OCLN*, was high in INF and it decreased toward PM (*P* < 0.05). *KRT5* was expressed at much lower level and only in INF (*P* < 0.05). As for progenitor markers, *SOX9* was uniformly expressed at high level across all fragments of the oviduct in hen (*P* < 0.05). Expression of *MSI1* and *CD44* was the highest in INF and it gradually decreased toward PM (*P* < 0.05). Expression of *NANOG* was detected, but was not significant (*P* > 0.05).

In quail oviduct (Fig. 3b), we had to use chicken primer sequences to show the expression pattern of *OVAL*, *OVM*, *KRT5*, *KRT14*, and *OCT4*/*cPOUV*. We have determined a similar pattern of the gene expression for two oviduct markers *OVAL* and *OVM,* which were expressed in all the studied oviduct compartments (INF, DM and PM). Whereas, oviduct marker for *ESR1* was significantly expressed in INF and DM compartments of a quail oviduct (*P* < 0.05). Among epithelial markers, the expression of *KRT14* and *KRT5* was high and increased toward INF, but expression of *OCLN* did not reach the significance threshold. In quail, we found significant expression of as much as four progenitor markers (*LGR5*, *OCT4*/*cPOUV, SOX9*, and *CD44*) (*P* < 0.05). *LGR5* and *OCT4*/*cPOUV* were most abundant in INF and DM compartments of the quail oviduct. Expression of *NANOG* was detected but it was not significant (*P* > 0.05).

### Gene profiling of the gene expression signatures in COEC and QOEC

**Fig. 4:**
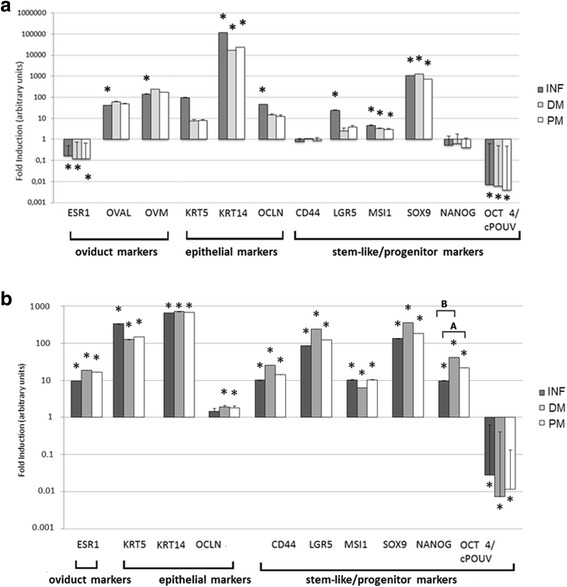
Expression of oviduct, epithelial, and progenitor markers in chicken (**a**) and quail (**b**) oviduct epithelial cells. Relative gene expression analysis was performed with RT-qPCR method in three oviduct fragments: infundibulum (INF), distal magnum (DM) and proximal magnum (PM). Pairwise t-test was conducted to determine the significant modulation of the gene expression in the oviduct as compared to the external calibrator (breast muscle) (*P* < 0.05). An asterisk (*) indicates that the gene is differentially expressed, compared to the calibrator. Letters A, B, and C in brackets indicate results of one-way ANOVA multiple comparisons between different fragments of the oviduct (*P* < 0.05)

After having determined gene expression signatures in three specific fragments of chicken and quail oviducts, we have established the respective cell cultures, which were analyzed for the presence of the same markers. Results of the relative gene expression analysis in chicken and quail oviduct epithelial cells are presented in Fig. 4a. In COEC (Fig. 4a) only few markers were numerically and significantly upregulated, namely *OVAL, OVM*, *KRT14*, and *SOX9* (*P* < 0.05). In the case of QOEC, we routinely found the abundance of ovalbumin in quail oviduct cell culture using antichicken OVA antibody and western blot detection. *MSI1* was upregulated statistically (*P* < 0.05), though it did not have high numerical values of fold induction. In both, COEC and QOEC, *OCT4/cPOUV* was significantly downregulated (*P* < 0.05). We did not determine any significant differences between COEC derived from different fragments of the oviduct, apart from the expression of *OCLN* (epithelial marker) and *LGR* (progenitor marker) that was high in the INF compartment (*P* > 0.05).

In QOEC, a similar significant expression, as in COEC, was found for progenitor markers S*OX9* and *MSI1* as well as epithelial marker *KRT14*. The remaining epithelial and stem-like/progenitor markers were significantly expressed in the cultivated quail cells derived from all studied compartments of oviduct (*P* > 0.05).

## Discussion

Among avian species, laying hen (*Gallus gallus domesticus*) and Japanese quail (*C. japonica*) provide two excellent experimental oviduct models to study the immunology and reproductive biology [20]. Particular properties of oviductal cells include hormonal regulators as well as biosynthetic and secretive activity, which can be used for biomedical applications. Firstly, the secreting function of an oviduct epithelium makes it an ideal natural bioreactor to obtain human therapeutic proteins by using genetic manipulation of the oviduct secretome. The product is accumulated in the egg white and is easily harvested [3]. Secondly, both hen and quail are recognized to reflect the development and chemoprevention of spontaneous leiomyoma, also known as fibroids of the oviduct in relation to human cancer [21]. Thirdly, the development of new oviduct cell lines would allow selectively propagating and studying important pathogens including *Campylobacter* and *Salmonella* strains or influenza and Coronaviruses. Such cell lines offer new in vitro substrates for pathogens originating from a reproductive tract.

For this purpose, we have attempted to provide a utility set of molecular markers to characterize the avian oviduct tissue in hen and quail and in vitro*-*derived oviduct epithelial cell culture. For a quail, only 900 proteins are annotated in the existing UniProt databases. Thus, when a gap in quail database [22] limits the interpretation of a sequence, a relevant genomic alignment onto the chicken is performed [23]. Depending on the database used (ENSEMBL, NCBi, and/or UniProt), sequences of the genes selected for this study had 89%–100% similarity. Thereby, gene expression assays developed were comparable between both species.

In our study, all 12 analyzed genes were expressed in both hen and quail. In the first part, we have characterized gene expression signatures in three compartments of the oviduct tissue in hen and quail. The mRNA abundance of the oviduct markers (*ESR1*, *OVAL*, and *OVM*) increased toward proximal parts of the oviduct. Those differences between infundibulum and magnum compartments were significant only in hen earlier, but we have determined a clear numerical pattern also in quail. Such a pattern of the oviduct markers reflects physiological functions of distinct compartments, e.g., oocyte transport and sperm storage in the infundibulum vs. egg white protein production in the magnum. For this reason, *ESR1*, which encodes the estrogen receptor 1, whose major function is binding estradiol—a major sex hormone of laying birds, was expressed in all parts of the oviduct. On the other hand, *OVAL* and *OVM*, which encode major egg white proteins, were expressed only in the magnum. Such a pattern of the gene expression across the avian oviduct has been widely reported in the literature [24, 25] and it validates the functional setup of this experiment.

In a panel of epithelial markers characterized *in tissue*, we have determined a reverse pattern, i.e., decrease of mRNA abundance from distal toward proximal parts of oviduct, in particular of *KRT14*, which was strongly expressed in the infundibulum of both, hen and quail. *KRT5* appeared to be more abundant in quail and *OCLN* was significantly expressed in chicken oviduct. Keratins encode for cytoskeletal proteins of highly proliferating basal epithelial cells [26]. Infundibulum is lined with ciliated epithelia, which are highly used by the frequent transportation of the oocyte and protein secretion. They require constant renewal from the basal epithelium, which is intensively proliferating. Strong induction of keratin genes is related with this function of the infundibulum. Previously, we have detected cytokeratins in chicken infundibulum by using immunohistochemistry technique, both *in tissue* and in vitro [6], which is in line with the results of the current study.

As for stem-like/progenitor markers analyzed *in tissue*, chicken expressed high mRNA abundance of *CD44* and *SOX9*; moderate abundance of *MSI1* and low of *NANOG*. *OCT4*/cPOUV and *LGR5* were not expressed in the chicken oviduct tissue. In quail, we determined a high-fold induction of *LGR5* and *OCT4*/*cPOUV* and a moderate abundance of *CD44* and *SOX9*. CD44 is a cell surface glycoprotein and an established progenitor/stem-like cell marker in fallopian tube in mammals. CD44-positive cell population showed the capacity for clonal growth and differentiation into tubal epithelial cells, particularly in the distal region of the tube [15, 27]. We earlier showed a high immunochemical stain of CD44 in the distal oviduct of a hen [6]. SOX9 is a transcription factor in early epithelial lineage [28, 29]. It is involved in the organogenesis of different tissues and its main function is to maintain a population of undifferentiated somatic stem cells. SOX9 was recently announced as a novel cancer stem cell marker [30]. In our study, we consider this gene as a marker for precursor epithelial oviduct cells of avian species. Musashi-1 is expressed in intestinal crypts and human endometrium, where it maintains multipotent potential for epithelial cells emerging from Müllerian duct (precursor of oviduct in vertebrates) [31]. OCT4/cPOUV and NANOG are chicken stem cell markers [32, 33], while LGR5 is recognized as marker stem cells in tubal epithelia [14]. In our study, *LGR5* and *OCT4*/*cPOUV* were detected at high level in quail oviduct. Overall, the pattern of expression of progenitor markers supports the designation of distal oviduct compartments as the source of progenitor epithelial cells.

After being transferred to in vitro conditions, phenotypes of COEC and QOEC have changed in some aspects. Secretive potential of magnum-derived cells was retained as reflected by the expression of *OVM* and *OVAL* in COEC and *ESR1* in QOEC. Primary cultured cells, such as highly specialized oviduct epithelial cells, are prone to rapid differentiation in vitro. This way, they may easily lose their original phenotype, for example, the ability for protein secretion. On the other hand, INF-derived COEC gained secretive potential after being cultured in vitro, which was reflected by changing the downregulation of *OVM* and *OVAL* to upregulation of those genes as compared to donor tissues. Stimulation with estrogen was reported as necessary to maintain responsiveness of hen oviduct cells to this sex hormone [34]. However, in this experiment, neither the birds were stimulated with the estrogen prior to tissue harvesting, nor the cultivated cells were treated with estrogen, which might explain the lack of *ESR-1* mRNA in the cultivated COEC. Epithelial character of both COEC and QOEC was maintained, especially in *KRT14* (COEC) and other epithelial markers (QOEC) mRNA abundance.

Expression of progenitor markers of early epithelial lineage (*SOX9*, *MSI1*, and *LGR5*) in both oviduct epithelial cultures was determined. *LGR5* was significantly upregulated in cultivated cells, and has been proven to mark the stem cells in murine oviduct/fimbria [14]. Precursor character of certain populations of cultured cells allowed for their proliferation and differentiation in vitro. INF-derived COEC gained gene expression signatures of oviduct secretive cells (*OVM* and *OVAL*). Population of progenitor cells is required for the establishment of a primary cell culture [35]. In our study, we have confirmed progenitor gene expression signatures in proliferating cultures. Based on the morphological assessment, a subpopulation of the cultured cells displayed epithelial character of ciliated and secreting cells. But there was also a large subpopulation of differentiated mesenchymal and fibroblast-like forms in both COEC and QOEC, after passaging. With these observations, a stable oviduct epithelial cell line could be probably established from both in vitro models, with the prior purification of progenitor cells from the heterogeneous starting cell populations.

## Conclusion

In this study, we have characterized the expression of oviduct, epithelial, and stem/progenitor markers in the oviduct tissue and cell culture of two avian species, the hen and the quail. Analysis of the oviduct tissue and cultured cells allowed for characterizing the molecular makeup of those cells *in tissue*, in relation to the source of the oviduct compartment (infundibulum, distal magnum, and proximal magnum). Further analysis from in vitro*-*cultivated cells showed molecular pattern that was different from noncultivated oviduct cells. In conclusion, the analysis of tissue material revealed a gradual increase/decrease pattern in majority of the markers in both species. This pattern changed after those cells had been cultured in vitro. A progenitor marker, *OCT4*/*cPOUV* was strongly downregulated in both in vitro models, whereas the expression of *SOX9* and the epithelial marker *KRT14* were not changed compared to the calibrator (FC ~ 1). Cultivated hen cells (COEC) gained the expression of *LGR5* progenitor marker, which could indicate a shift toward a more specific epithelial progenitor cell type. These results can contribute to further research on creating new biological models from reproductive tissue and the characterization required to develop new avian cell lines.

## Abbreviations

ACTB: β-actin
*C. japonica*: *Coturnix japonica*
CD44: Cell surface glycoprotein
COEC: Chicken oviduct epithelial cells
Ct: Cycle threshold
DM: Distal magnum
DMEM-F12: Dulbecco’s Modified Eagles Medium-F12
ESR1: Estrogen receptor 1
FBS: Fetal bovine serum
FC: Fold Change
*G. gallus*: *Gallus gallus*
GDP/GTP exchange factor VAV3: Guanyl nucleotide exchange factor
HMG: High mobility group box transcription factor
INF: Infundibulum
ITS: Insulin-Transferrin-Selenium
KRT14: Keratin 14
LGR5: Leucine-rich repeat containing G protein-coupled receptor 5
MSI-1: Musashi-1
NANOG: Nanog homebox
OCLN: Occludin
OCT4/cPOUV: Octamer-binding protein 4
OVAL: Ovalbumin
OVM: Ovomucoid
PM: Proximal magnum
QOEC: Quail oviduct epithelial cells
RT-qPCR: Reverse transcription - quantitative polymerase chain reaction
SEM: Standard error of the mean
SOX9: Sex determining region Y-box 9
UB: Ubiquitin C
